# Psychiatric symptoms of patients with primary mitochondrial DNA disorders

**DOI:** 10.1186/1744-9081-8-9

**Published:** 2012-02-13

**Authors:** Gabriella Inczedy-Farkas, Viktoria Remenyi, Aniko Gal, Zsofia Varga, Petra Balla, Agnes Udvardy-Meszaros, Benjamin Bereznai, Maria Judit Molnar

**Affiliations:** 1 Clinical and Research Center for Molecular Neurology, Department of Neurology, Semmelweis University, 1083 Budapest Tömő Str. 25-29., Budapest, Hungary; 2 Operative Clinical Department Medical Division, Chemical Works of Gedeon Richter Ltd., Budapest, Hungary; 3 Department of Psychiatry, Medical School and Health Science Center, University of Debrecen, Debrecen, Hungary; 4 Department of Clinical Psychology, Semmelweis University, Budapest, Hungary

**Keywords:** Mitochondrial mutation, Mental disorders, Depression

## Abstract

**Background:**

The aim of our study was to assess psychiatric symptoms in patients with genetically proven primary mutation of the mitochondrial DNA.

**Methods:**

19 adults with known mitochondrial mutation (MT) have been assessed with the Stanford Health Assessment Questionnaire 20-item Disability Index (HAQ-DI), the Symptom Check List-90-Revised (SCL-90-R), the Beck Depression Inventory-Short Form (BDI-SF), the Hamilton Depression Rating Scale (HDRS) and the clinical version of the Structured Clinical Interview for the the DSM-IV (SCID-I and SCID-II) As control, 10 patients with hereditary sensorimotor neuropathy (HN), harboring the peripheral myelin protein-22 (PMP22) mutation were examined with the same tools.

**Results:**

The two groups did not differ significantly in gender, age or education. Mean HAQ-DI score was 0.82 in the MT (range: 0-1.625) and 0.71 in the HN group (range: 0-1.625). Level of disability between the two groups did not differ significantly (*p *= 0.6076). MT patients scored significantly higher on the BDI-SF and HDRS than HN patients (12.85 versus 4.40, *p *= 0.031, and 15.62 vs 7.30, *p *= 0.043, respectively). The Global Severity Index (GSI) of SCL-90-R also showed significant difference (1.44 vs 0.46, *p *= 0.013) as well as the subscales except for somatization. SCID-I interview yielded a variety of mood disorders in both groups. Eight MT patient (42%) had past, 6 (31%) had current, 5 (26%) had both past and current psychiatric diagnosis, yielding a lifetime prevalence of 9/19 (47%) in the MT group. In the HN group, 3 patients had both past and current diagnosis showing a lifetime prevalence of 3/10 (30%) in this group. SCID-II detected personality disorder in 8 MT cases (42%), yielding 3 avoidant, 2 obsessive-compulsive and 3 personality disorder not otherwise specified (NOS) diagnosis. No personality disorder was identified in the HN group.

**Conclusions:**

Clinicians should be aware of the high prevalence of psychiatric symptoms in patients with mitochondrial mutation which has both etiologic and therapeutic relevance.

## Background

Mitochondrial disorders are metabolic conditions with chronic deterioration and multiple organ involvement. Primary mutations of the mitochondrial DNA (mtDNA) are inherited maternally while mitochondrial diseases due to mutations in nuclear DNA are transmitted as mendelian traits. The ratio of heteroplasmy (ratio of the mutant and wild type mtDNA) and threshold level (the proportion of mutated mitochondria required to cause dysfunction) varies from tissue to tissue, resulting in a wide variety of clinical phenotypes [1]. Some presentations of mtDNA mutations are clustered into syndromes such as MELAS (mitochondrial encephalomyopathy lactic acidosis and stroke-like episodes) or CPEO (chronic progressive external ophthalmoplegia), but most of them show great heterogeneity. In the background of the late onset and the progression of some mtDNA disorders there is good evidence for increases in the proportion of some pathogenic mutations - including pathogenic large scale deletions and tRNA point mutations - with age in skeletal muscle, however this is not a general phenomenon [2]. The inability to increase ATP production at times of higher energy demand explains why clinical symptomatology typically occurs in association with physiological [3] or psychological [4] stressors.

Brain tissue depends to a large extent on mitochondrial function for its metabolism, including the maintenance of the transmembrane potential [5], calcium homeostasis [6], signal transduction [7] and synaptic plasticity [8]. Therefore, impairments in oxidative phosphorylation tend to manifest in neurologic, psychiatric or neuropsychologic symptomatology [9].

There is a growing number of evidence for the association of mitochondrial dysfunction and psychiatric illnesses both in vitro and in vivo. Morphological changes [10], altered cellular location [11], decreased number [12] and function [13] of mitochondria has been found in diverse psychiatric conditions. Downregulation of mtDNA genes [14,15], reduced expression profiles of electron transport chain subunits [3,15], impaired defense against oxidative stress [16] and an overall dysfunction of brain metabolism at mitochondrial level [17] has also been described in association with psychiatric disorders.

While many case report suggests the new era of 'mitochondrial psychiatry' [18], a PubMed search yielded only one study where psychiatric assessment has been carried out systematically with an adult patient population selected by the presence of major and minor criteria of mitochondrial disease [19]. The aim of our study was to assess psychiatric symptoms in a cohort of patients with genetically proven primary mtDNA mutations which to our knowledge has not yet been performed.

## Methods

### Patients

Nineteen patients with primary mutation of the mtDNA were evaluated (MT patients, Patient 1-19, 13 female, 6 male). Mean age for the 13 probands, included in the statistical analysis was 34 ± 8.43 years (male: μ = 27.25, σ = 6.8; female: μ = 36.66, σ = 7.65), average years of education was 12.84 ± 1.21 years (male: μ = 13.25, σ = 1.5; female: μ = 12.66, σ = 1.12). As control, 10 patients (HN patients, Patient 20-29, 4 female, 6 male, mean age: 40 ± 10.99, average years of education: 14.2 ± 3.85 years) with hereditary sensorimotor neuropathy were examined. Diagnosis was based on clinical features supported by the presence of primary mtDNA mutations in MT patients and by the presence of the PMP22 gene mutation in HN patients.

All participants of the study were Caucasian and visited the clinic within 1 year. Written, informed consent was obtained from all participants. This study was carried out according to the Helsinki Declaration and was approved by the Research and Ethics committee of Semmelweis University.

### Neurological and psychiatric assessment

Neurological assessment was carried out with all MT and HN patients. Functional ability was assessed using the Hungarian validated version of the Stanford Health Assessment Questionnaire 20-item Disability Index (HAQ-DI) [20]. Detailed psychiatric assessment used the Symptom Checklist-90-Revised (SCL-90-R) [21], the Beck Depression Inventory-Short Form (BDI-SF) [22] self report inventories as well as the clinician-administered 21-item Hamilton Depression Rating Scale (HDRS) [23] and the clinical version of the Structured Clinical Interview for the DSM-IV axis-I (SCID-I) [24] and axis-II disorders (SCID-II) [25].

The SCL-90-R helps evaluate a broad range of psychopathological symptoms. It yields 9 scores of primary symptom dimensions (somatization, obsession-compulsion, interpersonal sensitivity, depression, anxiety, hostility, phobic anxiety, paranoid ideation and psychoticism), an additional item subscale referring to sleep and memory problems and the arithmetic mean of all of the above, the global severity index (GSI). The BDI-SF is a 13-item questionnaire scored on a 4-point scale, from 0 to 3, with overall scores ranging from 0 to 39. The BDI-SF has been found to have a good correlation with the standard 21-item BDI (r = 0.96, *p *= 0.001) and relates the clinical depth-of-depression (r = 0.61) [22]. The emphasis of the 21-item HDRS is on melancholic and physical symptoms of depression. In order to control for the confounding effect of cognitive impairment, we included patients with an IQ of 70 and above, as assessed by the Hungarian validated version of the Wechsler Adult Intelligence Scale (WAIS-III-R version). Scales and interviews were administered by trained clinicians. Patient charts were also reviewed.

### Statistical analysis

The Shapiro-Wilk test was employed to test for normality of the data (data not shown). Mann-Whitney *U*-test was performed, p-values were corrected by the Holm-Bonferroni method. All tests were two tailed and *p *values ≤ 0.05 were deemed significant. Differences between the two groups were assessed using Chi-Square test (gender) and *t*-test (age and education). SCL-90-R was analysed with SAS System for Windows (Release 9.1 TS Level 1 M3, Statistical Analysis System, SAS-Institute USA). Correlation of total scores in GSI, BDI and HDRS with HAQ-DI in both groups was evaluated using Pearson's correlation. Independent variables could be obtained selecting only the proband (index case) from each family from the cohort of MT patients. HN patients were all unrelated, statistics were thus performed using data from 13 MT (Patient 1, 2, 3, 5, 8, 10, 11, 13, 14, 15, 16, 18, 19) and 10 HN patients.

### Genetic analysis

DNA was extracted from blood cells. In cases with high suspicion of mitochondrial dysfunction where no mutation was found in blood cells, skeletal muscle biopsy was performed by Qiagen DNA extraction kit according to the manufacturer's instructions and previously published method [26]. Samples were screened at the most frequently mutated sites ('hot spots', mtDNA 'common mutations'; A3243G, A8344G, A8356G, T8993C, T8993G) with PCR-RFLP using standard methods. For the mtDNA 'common deletion' (a 4977 base-pair deletion between nucleotides 8470 and 13447), long-PCR methodology was used. In cases where no common mutation was found, the entire mtDNA was sequenced using standard methods. The PMP22 deletion and duplication was detected with real time PCR as published earlier [27]. The 5HTTLPR (serotonin-transporter-linked polymorphic region) genotypes of both MT and HN patients were detected by previously reported method [28].

## Results and Discussion

### Genotypes and clinical assessment

The two groups did not differ significantly in gender (Chi-square value = 1.9652; *p *value = 0.1610), age (t-value = -1.42; *p *value = 0.1711) or education (t-value = -1.20; *p *value = 0.243). Ten MT patients had common mutation of mtDNA. Four of them (Patient 1-4) harbored the A3243G base substitution in the tRNA^Leu ^resulting in MELAS syndrome (mitochondrial encephalopathy, lactic acidosis, stroke-like episodes), four patients (Patient 5-8) had the A8344G substitution in tRNA^Lys^. This mutation frequently causes MERRF (myoclonic epilepsy associated with ragged red fibers) syndrome. Patient 9 and 10 harbored the T8993G mutation in gene mitochondrial ATP synthase 6 gene which results in NARP syndrome (neuropathy, ataxia, retinitis pigmentosa), and four patients (Patient 11, 12, 13, 18) had common deletion of the mtDNA. MtDNA common deletion results in adult CPEO. Patient 14 had the A8332G base substitution in the tRNA^Lys ^and Patient 15 had the A12770G substitution resulting in a Glu - Gly substitution in gene ND5. In case of Patient 16 and 17, a C-T base substitution at nucleotide 14771 was present resulting in Pro-Ser substitution in CYB gene. In the case of Patient 19 - beside the A2706G polymorphism which is responsible for linezolid-induced severe lactic acidosis - 14 single nucleotide polymorphisms associated with Leber Hereditary Optic Neuropathy (LHON) were present potentially exerting synergistic effect.

Medication for the mitochondrial disease comprised of Coenzyme Q10, Vitamin E and vitamin C [29]. Some patients were taking psychiatric drugs at the time of the assessment. Monotherapies were clonazepam (Patient 7, 10, 14), sertraline (Patient 12), mirtazapine (Patient 13), aripiprazole (Patient 19), combination therapies were quetiapine and trazodone for Patient 16, clonazepam, sertraline and quetiapine for Patient 18. Anticonvulsant treatment was valproate for Patient 7 and 8, carbamazepine for Patient 14 and lamotrigine for Patient 16.

MtDNA and 5HTTLPR genotypes, clinical symptoms and medication of the MT group are summarized in Table 1.

**Table 1:** MtDNA and 5TTLPR genotypes, clinical symptoms and medication of MT patients

ID	mtDNA mutation	5HTTLPR genotype	Symptoms	Medication (with total daily dose)
*1*	*A3243G MELAS*	*L/S*	*Hypoacusis, ataxia, myopathy, endocrine dysfunction*	-

*2*	*A3243G MELAS*	*L/S*	*Ataxia*	-

*3*	*A3243G MELAS*	*L/S*	*CPEO, myopathy (mother of Patient 4)*	-

4	A3243G MELAS	L/S	CPEO	-

*5*	*A8344G MERRF*	*L/L*	*Migraine*	-

6	A8344G MERRF	S/S	Mild tremor in the upper limbs (mother of Patient 7 and 8)	-

7	A8344G MERRF	S/S	Myoclonus epilepsy, thrombocytopenia (twin brother of Patient 8)	levetiracetam (1000 mg), clonazepam (2 mg), valproate (1500 mg), vinpocetine (20 mg)

*8*	*A8344G MERRF*	*S/S*	*Myoclonus epilepsy, ataxia, cognitive dysfunction, thrombocytopenia (twin brother of Patient 7)*	*levetiracetam (2500 mg), valproate (2100 mg), vinpocetine (90 mg), trimetazidine (40 mg), betaxolol (10 mg), atorvastatine (10 mg), allopurinol (200 mg)*

9	T8993G NARP	L/S	Developmental abnormality of the right arm, hypothyreosis, (mother of Patient 10)	-

*10*	*T8993G NARP*	*L/L*	*NARP*	*clonazepam (1mg)* *vinpocetine(10 mg)*

*11*	*mtDNA del*	*L/L*	*CPEO, iron-deficient anemia, hypercholesterolemia*	-

12	mtDNA del	L/S	Myalgia, exercise intolerance	sertraline (50 mg)

*13*	*mtDNA del*	*L/S*	*Kearns-Sayre syndrome*	*clonazepam (1mg)* *vinpocetine (20 mg)* *mirtazapine (30mg)*

*14*	*A8332G* *C8270T*	L/S	*Dystonia, early onset stroke-like symptoms*	*levetiracetam (1000mg),clonazepam (1.25mg)* *carbamazepine (1000mg)*

*15*	*A12770G*	*L/S*	*Ataxia, hypoacusis, spastic paraparesis*	-

*16*	*A15326G* *A750G* *A1438G* *A8860G * *A15326G*	*L/L*	*Hypoacusis, limb tremor, myalgia, apraxia(mother of Patient17)*	*trimetazidine (60 mg)*, *metformin (850 mg*), *bisoprolol (5mg)*, *quetiapine (400 mg)*, *lamotrigine (50 mg)*, *trazodone (300mg)*

17	see Patient 16.	L/L	-	-

*18*	*A3720G* *G3849A* *T13020C* *T13734C* *A12308G* *T8473C*	*L/S*	*Severe cardiomyopathy, cognitive dysfunction*	*trimetazidine (40 mg), enalapril (5 mg), molsidomine (4 mg), quetiapine (400 mg), lamotrigine (100 mg), sertraline (100 mg), clonazepam (1.5 mg)*

*19*	*C14766T* *G709A* *A73G* *T16126C*	*S/S*	*Peripheral neuropathy*	*tolperisone (300 mg), aceclofenac (200 mg), aripiprazole (15 mg), duloxetine (30 mg), clonazepam (1 mg)*

Italics indicate probands (unrelated patients; independent variables) included in the statistical analysis

Abbreviations; *mtDNA del *deletion of the mtDNA. *MELAS *mitochondrial encephalopathy, lactic acidosis, stroke-like events; *CPEO *chronic progressive external ophthalmoplegia; *MERRF *myoclonic epilepsy with ragged red fibers; *NARP *neuropathia, ataxia, retinitis pigmentosa. 5HTTLPR: serotonin-transporter-linked polymorphic region; S/S genotype: short-short homozygous; L/L genotype: long-long homozygous; L/S genotype: long-short heterozygous

In the HN group, 9 patients harbored a duplication (Charcot-Marie-Tooth phenotype, CMT), and one patient had a deletion (Hereditary Neuropathy with Liability to Pressure Palsy phenotype, HNPP) in the PMP22 gene.

PMP22 and 5HTTLPR genotypes, clinical symptoms and medications of the HN group are summarized in Table 2.

**Table 2:** PMP22 and 5HTTLPR genotypes, clinical symptoms and medication of HN patients (control group)

ID	PMP22 mutation	5HTTLPR genotype	Symptoms	Medication (with total daily dose)
20	PMP22 duplication	L/L	Mild atrophy of the hand and feet muscles	-

21	PMP22 duplication	L/L	Pes equinovarus, moderate paresis in the distal muscles of the legs	-

22	PMP22 deletion	S/S	Sensory disturbancies in the lower limbs, neuropathy to pressure palsy	-

23	PMP22 duplication	S/S	Generalized muscle weakness, walking difficulty, distal type hypesthesia in the limbs, excavated feet	pregabalin (300 mg) duloxetine (30 mg)

24	PMP22 duplication	L/S	Paresthesia, distal type hypesthesia in the limbs, muscle cramps, gait instability	-

25	PMP22 duplication	L/S	Distal type muscle weakness in the limbs, tremor in the hands	chlordiazepoxide (5 mg) tolperisone (150 mg) propranolol (40 mg)

26	PMP22 duplication	L/S	Excavated feet, moderate distal paresis of the limbs, ataxia	-

27	PMP22 duplication	S/S	Mild paresis in the hand and feet muscles	-

28	PMP22 duplication	S/S	Moderate distal weakness of the limbs, distal type hypesthesia, head tremor, gait instability. Associated disorders: diabetes mellitus, hyperlipidaemia	duloxetine (60 mg) gabapentin (600 mg) rosuvastatine (20 mg) ezetimibe (10 mg)

29	PMP22 duplication	L/L	Moderate atrophy and paresis in the hand and feet muscles, gait instability. Associated disorder: asthma bronchiale	-

Abbr.: *PMP22 *peripheral myelin protein-22; 5HTTLPR: serotonin-transporter-linked polymorphic region; S/S genotype: short-short homozygous; L/L genotype: long-long homozygous; L/S genotype: long-short heterozygous

Mean HAQ-DI score was 0.82 in the MT (range: 0-1.625) and 0.71 in the HN group (range: 0-1.625). Level of disability between the two groups did not differ significantly (*p *= 0.6076).

### Psychiatric assessment

Significant difference was found in the GSI score (1.44 vs 0.46, *p *= 0.013) and the nine subscales of the SCL-90-R scale. These subscales were obsession-compulsion (*p *= 0.0079), interpersonal sensitivity (*p *= 0.0079), depression (*p *= 0.0309), anxiety (*p *= 0.0309), hostility (*p *= 0.0428), phobic anxiety (*p *= 0.0309), paranoid ideation (*p *= 0.0101) psychoticism (*p *= 0.0002) and additional items (*p *= 0.013). No significant difference was found between the two groups' somatization score (*p *= 0.0817).

BDI-SF and HDRS scores of the two groups also differed significantly (12.85 vs 4.40, *p *= 0.0309, and 15.62 vs 7.30, *p *= 0.0428, respectively). No correlation has been found between total scores of GSI, BDI and HDRS with HAQ-DI either in the MT or in the HN group as assessed by Pearson's correlation.

Results of the SCL-90-R, BDI-SF, HDRS and HAQ-DI are summarized in Table 3.

**Table 3:** Comparison of SCL-90-R, BDI-SF, HDRS and HAQ-DI scores of MT and HN patients

	MT group		HN group		t-value	df	D	p value (Raw)	p value (Adjusted)
						
Measured items	mean	SD	mean	SD					
**GSI**	1.44	0.91	0.46	0.53	3.64	21	1.587	0.0015	0.0130*

**Somatization**	1.77	1.10	0.98	0.83	1.83	21	0.798	0.0817	0.0817

**Obsessive-compulsive**	1.65	1.04	0.47	0.68	3.99	21	1.743	0.0007	0.0079*

**Interpersonal sensitivity**	1.55	0.97	0.40	0.51	3.99	21	1.740	0.0007	0.0079*

**Depression**	1.90	1.21	0.75	1.03	3.12	21	1.362	0.0052	0.0309*

**Anxiety**	1.32	1.14	0.42	0.63	3.19	21	1.392	0.0044	0.0309*

**Hostility**	1.26	1.00	0.48	0.50	2.60	21	1.133	0.0169	0.0428*

**Phobia**	1.14	1.11	0.29	0.56	3.06	21	1.335	0.0060	0.0309*

**Paranoia**	1.42	1.05	0.28	0.39	3.81	21	1.665	0.0010	0.0101*

**Psychoticism**	0.92	0.60	0.13	0.24	5.65	21	2.465	<.0001	0.0002*

**Additional items**	1.48	0.96	0.43	0.66	3.66	21	1.599	0.0014	0.0130*

**BDI-SF**	12.85	8.33	4.40	5.36	3.18	21	1.386	0.0045	0.0309*

**HDRS**	15.62	8.62	7.30	5.52	2.67	21	1.166	0.0143	0.0428*

**HAQ-DI**	0.82	0.59	0.71	0.59	0.52	21	0.228	0.6076	0.6076

Abbr.: *SCL-90-R *Symptom Checklist-90-Revised; *GSI *global severity index; *BDI *Beck Depression Inventory-Short Form; *HDRS *Hamilton Depression rating Scale, *HAQ-DI *Stanford Health Assessment Questionnaire Disability Index. D: Cohen's d effect size, Adjusted_p: *p *values with Bonferroni-Holm correction. Asterisks refer to statistically significant differences (*p *≤ 0.05) between the two groups.

A variety of psychiatric disorders was diagnosed with SCID-I, with a current diagnosis in 6 (31%), past diagnosis in 8 (42%), both past and current diagnosis in 5 (26%), a lifetime prevalence in 9 (47%) MT cases. Past or current major depressive disorder, dysthymia, bipolar II and adjustment disorder with depressed mood occurred in 2 MT cases while the past or current diagnosis of major depression with psychotic features, bipolar I, mixed anxiety-depressive disorder, postpartum depression and PTSD was explored in 1 MT case.

Eight MT patients were diagnosed with personality disorder representing 42% of the group. Three cases of avoidant and 2 cases of obsessive-compulsive personality disorder were diagnosed. In 3 cases, personality disorder not otherwise specified (NOS) was described referring to depressive personality in case of Patient 5 and mixed personality disorder in the cases of Patient 16 and 18.

Five MT patients (Patient 1, 5, 8, 16, 18) had both Axis I and II diagnosis. This subgroup also had mean BDI and GSI scores above the average of the entire MT group (BDI of 19.2 versus 12.85 and GSI of 2.29 versus 1.44).

Patient 5, 6, 9, 11, 12, 17 was free of somatic symptoms (indicated with a HAQ-DI score of 0) but only Patient 6 and 12 was free of psychiatric symptoms as well (although Patient 12 was on Sertraline 50 mg at the time of the assessment for subclinical anxiety). From this physically asymptomatic group Patient 5 and 11 was included in the statistical analysis (independent variables). They have higher BDI (15.5 versus 12.85) and GSI (1.66 versus 1.44) compared to the entire MT group.

Gender, age, results of the SCID interviews, together with the BDI-SF, GSI and HAQ-DI scores of the MT group are indicated in Table 4.

**Table 4:** Gender, age, results of SCID-I and SCID-II, BDI-SF, GSI and HAQ-DI scores of MT patients

ID	Gender	Age	Past diagnosis **SCID I**.	**Current diagnosis SCID I**.	**Personality disorder SCID II**.	BDI	GSI	HAQ-DI
*1*	*F*	*34*	*Mixed anxiety-depressive disorder*	*Major depressive disorder*	*Avoidant*	*22*	*1.92*	*1.375*

*2*	*F*	*51*	*Dysthymia*	*-*	*-*	*8*	*1.09*	*0.875*

*3*	*F*	*34*	*-*	*-*	*-*	*8*	*1.09*	*1.25*

*4*	*F*	*16*	*-*	*-*	*-*	*14*	*1.24*	*0.125*

*5*	*M*	*32*	*-*	*Dysthymia*	*Personality disorder NOS*	*21*	*2.46*	*0*

6	F	61	-	-	-	0	0.33	0

7	M	34	Adjustment disorder with depressed mood	-	-	2	0.31	0.375

*8*	*M*	*34*	*Adjustment disorder with depressed mood*	*-*	*Avoidant*	*3*	*0.87*	*1.625*

9	F	59	-	-	Obsessive - compulsive	8	0.59	0

*10*	*M*	*23*	*-*	*-*	*-*	*7*	*0.44*	*1.375*

*11*	*F*	*34*	*-*	*-*	*-*	*10*	*0.86*	*0*

12	F	40	-	-	-	2	0.22	0

*13*	*F*	*38*	*Bipolar II disorder*	*Bipolar II, current episode depressive*	*-*	*16*	*1.19*	*0.375*

*14*	*M*	*20*	*-*	*-*	*-*	*4*	*0.78*	*0.125*

*15*	*F*	*22*	*-*	*-*	*Obsessive - compulsive*	*5*	*0.74*	*1.625*

*16*	*F*	*39*	*Major depression with psychotic features*	*Major depressive disorder*	*Personality disorder NOS*	*30*	*3.02*	*0.625*

17	M	20	-	-	Avoidant	2	0.25	0

*18*	*F*	*37*	*PTSD*	*Bipolar II, current episode depressive*	*Personality disorder NOS*	*20*	*3.2*	*0.625*

*19*	*F*	*41*	*Postpartum depression*	*Bipolar I. latest episode depressive*	*-*	*13*	*1.08*	*0.75*

Italics indicate probands (unrelated patients; independent variables) included in the statistical analysis.

Abbr.: *M *male, *F *female, *SCID *structured clinical interviews for the DSM-IV; Personality disorder NOS: personality disorder not otherwise specified; *BDI-SF *Beck Depression Inventory Short Form, *SCL-90-R *Symptom Checklist revised; *GSI *global severity index, *HAQ-DI *Stanford Health Assessment Questionnaire 20-item Disability Index; *PTSD *posttraumatic stress disorder

In the HN group, 3 patients (30%) had past and current psychiatric diagnosis. Two HN patients had lifetime prevalence of dysthymia, 1 HN patient had lifetime prevalence of major depression, bipolar II, adjustment disorder with depressed mood and alcohol abuse. No personality disorder was found. Somatically asymptomatic (HAQ-DI score of 0) HN patients were Patient 20 and 27 with a lower mean BDI and GSI than the HN group's average (0.5 versus 4.4 and 0.11 versus 0.46, respectively).

Gender, age, results of the SCID interviews, together with the BDI-SF, GSI and HAQ-DI scores of the HN group are shown in Table 5.

**Table 5:** Gender, age, results of SCID-I and SCID-II, BDI-SF, GSI and HAQ-DI scores of HN patients

ID	Gender	Age	Past diagnosis SCID I	Current diagnosis SCID I	Personality disorder SCID II	BDI	GSI	HAQ-DI
20	M	41	-	-	-	1	0.22	0

21	M	17	-	-	-	1	0.32	0.125

22	M	49	Adjustment disorder with depressed mood	Dysthymia	-	11	1.3	0.375

23	F	33	-	-	-	2	0.18	0.875

24	M	34	-	-	-	2	0.12	0.5

25	F	46	-	-	-	2	0.26	1.25

26	F	31	-	-	-	11	0.37	1.125

27	M	42	-	-	-	0	0	0

28	F	55	Major depressive disorder	Dysthymia	-	14	1.59	1.25

29	M	47	Alcohol abuse	Bipolar II, latest episode hypomanic	-	0	0.25	1.625

Abbr: M male, F female, SCID structured clinical interview for the DSM-IV; BDI-SF Beck Depression Inventory Short Form; SCL-90-R Symptom Checklist Revised; GSI global severity index, HAQ-DI Stanford Health Assessment Questionnaire 20-item Disability Index

## Discussion

This is the first systematic study investigating psychiatric symptoms in a well-defined cohort of patients with genetically proven mtDNA-related mitochondrial disorders compared to a homologous patient group with genetically determined debilitating neuromuscular disease. Comparable levels of disability are suggested by similar mean HAQ-DI and SCL-90-R somatization scores. Our data revealed a higher prevalence of psychiatric symptoms, especially various mood disorders in the MT group supporting the hypothesis that mitochondrial dysfunction of the central nervous system may play a role in the pathogenesis of different psychiatric spectrum disorders, including depressive disorders [30]. MtDNA point mutations [31] and deletions [32] have been previously associated with major affective disease. The MR-spectroscopy literature supports the presence of brain metabolic alterations in bipolar disorder with variable relationships to the mood state [33]. Mitochondrial dysfunction may alter the signaling of hippocampal neurons via calcium-dependent mitochondrial superoxides, modulating nuclear cAMP-responsive element-binding protein (CREB) phosphorylation [34], which may play an important role in mood modulation [35]. In our study both the BDI-SF and HDRS scores were significantly higher in the MT group. SCID-I interview in the MT group yielded a high prevalence of mood disorders as well. The fact that SCID-I did not detect mood disturbance in some patients with moderately high BDI-SF scores likely reflects that patients generally tend to admit more symptoms on a self-inventory questionnaire than on an interview. Also, despite distinguished screening and diagnostic cutoff scores for the BDI-SF [36] it is important to note that a high score can be added up from a few items and does not necessarily mean that the patient actually have depression. The BDI-SF includes cognitive-affective but not somatic items to avoid spuriously high scores and overreporting of depression in somatic patients [37]. Instruments measuring both cognitive, affective, somatic, and behavioral symptoms of depression are more accurate for diagnosing major depression [37]. The BDI-SF should be regarded as an initial screening instrument and a severity index of depression in patients with multisystemic symptomatology.

The short-short (s/s) homozygous length polymorphisms of the 5HTTLPR has been implied in the genetic background of depression [38]. Although the association is not straightforward [39] and has been questioned [40], the 5HTTLPR is still widely studied in the development of neuroticism and depression [41]. In our study, those having high levels of depression in the MT group did not harbor the s/s homozygous polymorphism.

Although the SCL-90-R is not a diagnostic scale, significant differences found between nine SCL-90-R subscale scores suggest that MT patients are more prone to a variety of psychiatric problems. Subscales with highly significant differences were obsessive-compulsive symptoms, interpersonal sensitivity and psychoticism.

Personality disorders were also highly represented in the MT group, the most prevalent was avoidant personality. Avoidant personality traits have been described with mtDNA mutations [42]. It possibly reflects the fact that some mitochondrial patients are symptomatic early in childhood predisposing them to peer rejection and its long-term psychological consequences. Avoidance can also become a survival skill when one is not able to keep up with peers, e.g., at running as fast as other children, or being more easily fatigued. Obsessive personality disorder was present in 2 MT cases. In a previous report a case of MELAS syndrome coexisting with obsessive-compulsive disorder was presented where the patient had a 'perfectionistic and obsessional' premorbid personality [43]. The highly significant (*p *= 0.0079) SCL-90-R obsessive-compulsive subscale score implies that MT patients are generally more prone to obsessive-compulsive symptomatology. Eight of our 19 MT patients were diagnosed with personality disorder which is a high prevalence (42%). Those diagnosed with both axis I and II disorder also scored higher on the BDI and GSI. Relevant literature is scarce; mostly 'organic personality change' - due to the gradual cognitive and emotional decline - has been described [17]. Further studies are needed to clarify the association between the pathomechanism of personality disorders and mitochondrial cytopathies.

Although correlation between somatic and psychiatric symptoms was not found in either group, we demonstrated that physically asymptomatic HN patients score lower than their groups on the BDI and GSI, but not physically asymptomatic MT patients. It was also presented that the physically and psychiatrically asymptomatic subgroups of the MT cohort had little overlap. According to these results, psychiatric and somatic symptoms do not necessarily co-occur in the MT group implying that psychiatric symptoms might be independent manifestations of mitochondrial dysfunction and not the consequence of the chronic somatic disease. Psychiatric symptoms of these patients tend not to have a classic course [19]. They are frequently treatment-resistant and may even get worse when treated with psychotropic medications. Valproic acid, for instance, is known to interfere with beta-oxidation and is considered to be a mitochondrial toxin [44]. Drug-naivity of our patients could not be achieved due to ethical reasons. Despite diverse medication - also psychotropic medication - we depicted many psychiatric symptoms possibly due to treatment resistance, the complex metabolic alteration in the background.

## Conclusions

We detected a high prevalence of psychiatric symptoms in patients with mtDNA disorders. The early identification of these patients is important in order to avoid adverse effects of experimental psychotropic pharmacotherapy and to slow the progression of the mitochondrial disease by administering coenzyme Q10 and free radical scavengers. If the proband is diagnosed, maternal relatives and siblings can also be treated at an early phase. Further systematic studies are required to clarify the association of mitochondrial and psychiatric disorders and to improve diagnostics as well as therapeutics for these patients in the new era of personalized medicine.

## Limitations

Our study had several methodological limitations; we examined small number of patients because primary mtDNA-related mitochondrial diseases are relatively rare and often underdiagnosed. Due to this small patient number, statistical significances have limited strength. We examined families therefore statistical analysis could only involve probands to keep variables independent. Some of our patients were on psychiatric medication at the time of the assessment which could potentially bias our results. Medication was not included into statistics neither as variable due to the high diversity of drugs used nor as a factor with 2 levels because dividing these small patient groups into subgroups would have further weakened our results. The presented results may not generalize to all patients with mitochondrial disorders.

## Abbreviations

mtDNA: Mitochondrial DNA; MT: Patients with primary mtDNA mutation; MELAS: Mitochondrial encephalopathy: lactic acidosis: stroke-like episodes; MERRF: Myoclonic epilepsy associated with ragged red fibers; NARP: Neuropathy, ataxia, retinitis pigmentosa; LHON: Leber hereditary optic neuropathy; HN: Patients with PMP22 mutation; PMP22: Peripheral myelin protein-22; CMT: Charcot-Marie-Tooth phenotype of the PMP22 mutation; HNPP: Hereditary neuropathy with liability to pressure Palsy phenotype of the PMP22 mutation; 5HTTLPR: 5HT (serotonin)-transporter-linked polymorphic region; SCL-90-R: Symptom checklist-90-revised; GSI: Global severity index of the SCL-90-R; BDI-SF: Beck depression inventory-short form; HDRS: Hamilton depression rating scale; SCID-I and SCID-II: Structured clinical interviews for the DSM-IV; HAQ-DI: Stanford health assessment questionnaire 20-item disability index; NOS: Not otherwise specified.

## Competing interests

The authors declare that they have no competing interests.

## Authors' contributions

GIF and PB carried out psychiatric assessments, GIF analyzed and interpreted all data and drafted the manuscript. VR and AG carried out the molecular genetic studies. ZV carried out the statistical analysis, AM performed neuropsychological assessment and IQ testing. BB performed muscle biopsies and managed myopathological analysis of the patients. MJM recruited patients, performed neurological examinations, designed and conceptualized the study, analyzed and interpreted all data and drafted the manuscript. All authors read and approved the final version of the manuscript.

## Funding

The study was supported by the grant of Gabor Baross NEUPGX and TAMOP 4.2.1B-09/1/KMR-2010-001 to Gabriella Inczedy-Farkas, Viktoria Remenyi, Aniko Gal, Benjamin Bereznai and Maria Judit Molnar.
